# Short bowel syndrome presenting with re-feeding syndrome in a Han Chinese patient: a case report

**DOI:** 10.1186/1752-1947-6-137

**Published:** 2012-05-30

**Authors:** Ying Xie, Weiming Zhu, Ning Li, Jieshou Li

**Affiliations:** 1 Department of General Surgery, Jinling Hospital, Nanjing University School of Medicine, No 305 East Zhongshan Road, Nanjing 210002, P R China

## Abstract

**Introduction:**

Re-feeding syndrome is common in patients with long-term starvation. To the best of our knowledge this case is the first to report a patient with short bowel syndrome developing re-feeding syndrome 12 years after the bowel resection.

**Case presentation:**

A 33-year-old Chinese Han man underwent small bowel resection leaving only 40 cm of bowel, without an ileocecal valve, 12 years previously. At that time he was weaned from total parenteral nutrition and had a normal diet. He later developed features of severe malnutrition, and when parenteral nutrition was given, he developed re-feeding syndrome.

**Conclusion:**

Although re-feeding syndrome is a common complication in patients with any kind of nutritional support, and known to us for many years, high risk patients still need more attention and monitoring. Re-feeding syndrome in this case was not only a macronutrients deficiency but also a micronutrient deficient, and prompt supplement therapy and organ function support proved to be successful.

## Introduction

People are at great risk of re-feeding syndrome (RFS) when they are severely malnourished at the time of re-feeding. RFS usually occurs during the early stage of oral, enteral or parenteral feeding. To illustrate the awareness of RFS we report a case of RFS secondary to short bowel syndrome (SBS).

## Case presentation

A 33-year-old man was admitted to our hospital because of generalized weakness, emaciation and anorexia 12 years after massive small bowel resection. Our patient underwent massive small bowel and ileocecal resection because of small bowel volvulus 12 years ago. An end-to-side anastomosis was performed between his proximal jejunum and his ascending colon, with only 40 cm of his small bowel remaining. Subsequent bowel rehabilitation therapy, which included enteral nutrition with dietary fiber, recombinant human growth hormone and glutamine, was instituted for four weeks [1]. Our patient was weaned from total parenteral nutrition (TPN) and had a normal diet up to the present.

One month ago, our patient developed diarrhea and weight loss of 7 kg with no obvious predisposing cause; he felt weak, anorexic and dizzy, and became progressively cachexic. Urinary volume was reduced, while stool volume was increased. On admission, our patient's body weight was 36 kg, body mass index (BMI) 12.2 kg/m^2^, heart rate 80 beats per minute (bpm), blood pressure 70/50 mmHg, and respiratory rate 18 bpm. He was oriented and cognitively intact. Pitting edema appeared in his lower extremities. An abdominal examination revealed previous surgery scarring, thin skin, a scaphoid abdomen, and negative shifting dullness. A chest X-ray showed a high density shadow of a nodule outside his right upper lung, considered to be rib calcification. A lower limb X-ray and ultrasound of both kidneys and ureters were normal. An ultrasound of his gallbladder showed gallbladder atrophy. An electrocardiogram showed normal sinus rhythm with T wave changes (II/III/avF/v4-6 flat, inversion), with incomplete right bundle branch block. All laboratory values are presented in Table 1.

**Table 1:** Laboratory values

Lab Values	Normal	Initial	Day 3	Day 4	Day 5	Day 6	Day 7	Day 8	Day 9	Day 18
Phosphate, mmol/L	0.7-1.5	1.10	0.25	0.05	0.09	0.50	1.10	1.31	0.91	1.73
Potassium, mmol/L	3.5-5.5	3.1	3.1	4.9	5.5	3.9	3.2	3.4	4.2	4.8
Sodium, mmol/L	136-148	144	145	145	144	135	138	148	147	144
Glucose, mmol/L	3.9-6.2	6.0	3.5	7.7	5.2	8.9	5.2	6.3	6.8	5.0
Magnesium, mmol/L	0.6-1.0	0.60	0.57	0.76	0.73	0.50	0.60	0.66	0.92	0.91
Calcium, mmol/L	2.1-2.6	1.7	2.1	2.0	2.3	2.0	2.1	2.3	2.3	2.4
Albumin, g/L	35-55	27.7	27.6	30.0	32.4			32.3	31.4	37.7
Prealbumin, mg/dL	250-400		100	161	196			125	116	209
Hemaglobin, g/L	120-160	48	62	97	100	104	102	84	97	98
Hematocrit, %	45-50	14.0	18.1	28.6	30.5	31.5	30.3	25.5	28.6	29.8
blood urea nitrogen, mmol/L	2.9-7.5	5.0	5.2	2.8	6.9	6.0		8.4	11.1	9.8
Creatine, mg/dL	45-110	86	68	86	67	49		152	70	46

Our patient was diagnosed with severe malnutrition and was supported by blood transfusion, parenteral nutrition (500 kcal/day), fluid, electrolyte, trace element and multi-vitamin supplementation (one ampoule each of Soluvit^® ^and Addamel^® ^daily), along with thiamine 100 mg intravenously prophylactically. Our patient's physical strength improved after these treatments, and pitting edema of the lower extremities decreased. Within 24 hours, our patient presented with profound fatigue and flaccid weakness of all four limbs. His heart rate was 98 bpm, respiration was normal, but biochemical investigation showed severe hypophosphatemia 0.05 mmol/L (Table 1), hence a clinical diagnosis of RFS was made, and immediately the patient was transferred to the intensive care unit (ICU) for closer monitoring. Parenteral and enteral nutrition were interrupted and intravenous supplementation with phosphate polyfusor (120 mmol on the first day, then 60 mmol per day intravenously or enterally for five days) was initiated, along with enteral and parenteral multi-vitamins and trace elements (two ampoules each of Soluvit^®^, Vitalipid^® ^and Addamel^® ^daily for two days, thereafter one ampoule of each daily). He also received a once daily intramuscular dose of Vitamin B12 0.1 mg and thiamine 100 mg. In addition, a hydrocortisone injection 100 mg was given to prevent adrenal insufficiency on the fifth day. A plasma transfusion was given to expand blood volume because blood pressure fell to 97/60 mmHg temporarily.

On the seventh day, our patient had numbness of his limbs, diarrhea and dyspnea. At the same time, a chest X-ray indicated right middle lobe pneumonia. Respiratory muscle weakness was considered to be the reason, and an emergency tracheotomy with mechanical ventilation was done, and sputum aspiration was regularly performed. Anti-diarrhea (diphenoxylate 1 pill three times a day by mouth), anti-infection (moxifloxacin 0.4 g/day intravenously), and antacid (omeprazole 40 mg intravenously twice daily) therapy were given immediately.

On the ninth day, when his blood chemistry became normal, enteral nutrition was resumed at 500 kcal/day and increased gradually. On the twelfth day, total enteral nutrition was well tolerated with improved pulmonary symptoms, and our patient was successfully weaned from mechanical ventilator support. Subsequently, he received recombinant human growth hormone therapy (10 u/d) and total enteral nutritional support for one month and recovered uneventfully with a weight of 45 kg (gain of 9 kg) and a BMI of 15.2 kg/m^2^.

## Discussion

RFS was first recognized in post-World War II prisoners [2]; the exact incidence is unknown, but extremely malnourished patients are a susceptible population [3]. It is reported that 25% of cancer patients receiving TPN present with hypophosphatemia [4,5], and in severely malnourished cancer patients, the incidence of hypophosphatemia may go up to 100% [6].

The clinical presentation of re-feeding syndrome is characterized by fluid and electrolyte disturbance, which is caused by a shift of electrolytes and vitamins from extracellular space during starvation to intracellular space after re-feeding with glucose and amino acids [3]. Cellular uptake of glucose and amino acids during anabolism increases insulin secretion, causing increased cellular uptake of phosphate, potassium, magnesium and thiamine. This leads to a dramatic depletion of serum levels of these elements, which are the laboratory findings of re-feeding syndrome. Hypophosphatemia appears to be the predominant manifestation of RFS [7].

Re-feeding syndrome can be asymptomatic during the prodromal stage; as a result, it may be overlooked if the physicians are not aware of the risk or do not evaluate the electrolyte levels frequently [8]. Besides abnormalities in serum levels of glucose and electrolytes, RFS patients may also manifest symptoms from various systems, including the cardiovascular, pulmonary, neuromuscular and hematologic systems [3,9].

Re-feeding syndrome is diagnosed by a history of severe malnutrition and re-feeding, the clinical symptoms of cardiovascular, respiratory, neurological or skeletomuscular systems, hypo- or hyperglycemia, and the abnormalities of fluid and electrolytes [4,7,10,11]. A differential diagnosis should be considered; including hemodialysis, diabetic ketoacidosis, sepsis, volume repletion, malabsorption and multiple medication usage, but treatment must be initiated early because of the high mortality rate after delayed diagnosis. Repletion of electrolytes (including trace elements) and multi-vitamins (including thiamine, vitamin B12) intravenously to maintain a normal blood level of these elements must be initiated early, before nutritional support can be started. A simple supplementation of individual elements (for example, phosphorus or potassium) or correction of hypoglycemia is not effective. Symptomatic treatment to support the vital signs, such as tracheotomy and mechanical ventilation, is also important for the treatment success in the patient.

Because of the high mortality associated with RFS, assessing risk and an early awareness of RFS in malnourished patients is much more important than treatment after symptom attack [12]. For the high risk patient, nutrition should be given after sufficient supplementation with electrolytes (including trace elements) and multi-vitamins (including thiamine), and should be initiated at a low calorie level (about 30% of requirements), and slowly increased.

This case illustrates the vulnerability of SBS patients with rapid weight loss and an extremely low BMI to RFS. This patient had only 40 cm of small bowel with no ileocecal valve remaining, which predisposed him to severe malnutrition. Although serum levels of electrolytes were normal on admission, our patient developed RFS after initiation of parental nutrition, which switched his metabolic state from low catabolism to anabolism. A rapid shift of electrolytes (especially phosphate P^+^, potassium K^+ ^and magnesium Mg^2+^) occurred from plasma to intracellular space, leading to a sharp decrease of plasma levels of these elements. Our patient also suffered hypotension, dyspnea, pneumonia, flaccid paralysis and lethargy during the course of the disease. Parental nutrition was withdrawn immediately after the onset of the symptoms, and a sufficient amount of electrolytes and multi-vitamins were initiated intravenously and promptly based on a careful monitoring of their levels. Our patient was transferred to the intensive care unit, thus ensuring close monitoring. The key management was emergency tracheotomy with mechanical ventilation to prevent hypoxemia and acute respiratory failure.

## Conclusion

Clinicians should still be aware of the high risks and complications of RFS, whether the nutritional support is oral, enteral, or parenteral. Special attention should be paid to the early correction of not only macronutrients (potassium, phosphate, magnesium and thiamine), but also micronutrients like trace elements and vitamins, before nutritional support can be initiated. Organ function support should be initiated without hesitation once any symptom or sign of organ dysfunction emerges. Early monitoring and prophylactic supplement could prevent RFS. Although prompt supportive management could reverse the deterioration, careful recognition of patients at risk is still crucial for clinicians and the nutrition team.

## Abbreviations

bpm: beats per minute; RFS: refeeding syndrome; SBS: short bowel syndrome; TPN: total parenteral nutrition.

## Consent

Written informed consent was obtained from the patient for publication of this case report and any accompanying images. A copy of the written consent is available for review by the Editor-in-Chief of this journal.

## Competing interests

The authors declare that they have no competing interests.

## Authors' contributions

XY analyzed and interpreted the patient data regarding the history of surgery and the experience with this disease. ZWM performed the previous surgery and conducted the bowel rehabilitation therapy. LN diagnosed the disease and gave the correct therapy. LJS revised this manuscript critically. All authors read and approved the final manuscript.
